# A Single Question of Parent-Reported Physical Activity Levels Estimates Objectively Measured Physical Fitness and Body Composition in Preschool Children: The PREFIT Project

**DOI:** 10.3389/fpsyg.2019.01585

**Published:** 2019-07-10

**Authors:** Pere Palou, Adrià Muntaner-Mas, Jaume Cantallops, Pere Antoni Borràs, Idoia Labayen, David Jiménez-Pavón, Cecilia Dorado García, Diego Moliner-Urdiales, Manuel A. Rodríguez Pérez, Miguel A. Rojo-Tirado, Cristina Cadenas-Sanchez, Francisco B. Ortega, Josep Vidal-Conti

**Affiliations:** ^1^GICAFE “Physical Activity and Exercise Sciences Research Group”, University of the Balearic Islands, Palma, Spain; ^2^PROFITH “PROmoting FITness and Health Through Physical Activity” Research Group, Faculty of Sport Sciences, Department of Physical Education and Sports, University of Granada, Granada, Spain; ^3^Institute for Innovation & Sustainable Development in Food Chain (IS-FOOD), Public University of Navarre, Pamplona, Spain; ^4^MOVE-IT Research Group and Department of Physical Education, Faculty of Education Sciences, University of Cádiz, Cádiz, Spain; ^5^Biomedical Research and Innovation Institute of Cádiz (INiBICA) Research Unit, Puerta del Mar University Hospital University of Cádiz, Cádiz, Spain; ^6^Research Institute of Biomedical and Health Sciences (IUIBS), University of Las Palmas de Gran Canaria, Las Palmas de Gran Canaria, Spain; ^7^Department of Physical Education, University of Las Palmas de Gran Canaria Las Palmas de Gran Canaria, Spain; ^8^LIFE Research Group, University of Jaume I, Castellón de la Plana, Spain; ^9^Faculty of Education Sciences, SPORT Research Group, CERNEP Research Center, University of Almería, Almería, Spain; ^10^Laboratory of Exercise Physiology Research Group (LFE Research Group), Faculty of Physical Activity and Sport Sciences-INEF, Department of Health and Human Performance, Universidad Politécnica de Madrid, Madrid, Spain

**Keywords:** physical fitness, motor activity, body composition, fatness, physical activity, preschool children

## Abstract

Physical inactivity is recognized as a determinant of low physical fitness and body composition in preschool children, which in turn, are important markers of health through the lifespan. Objective methods to assess physical activity, physical fitness and body composition in preschool children are preferable; however, they have some practical limitations in the school context. Therefore, the aim of this study was to test whether a single question regarding physical activity level of preschool children, reported by their parents, could be used as an alternative screening tool of physical fitness and body composition. The information was obtained from 10 different cities throughout Spain, gathering a total of 3179 healthy preschool children (52.8% boys and 47.2% girls) aged 3–5 years. Physical activity levels of preschool children were reported by parents using a single question with five response options (very low, low, average, high, or very high). Physical fitness and body composition were assessed with the PREFIT fitness battery. The results showed that parents’ perception of their children’s physical activity was positively associated with all objectively measured physical fitness components (β_range_ = -0.094 to 0.113; all *p* < 0.020); and negatively with body composition indicators as measured (β_range_ = -0.113 to -0.058; all *p* < 0.001). The results showed significant differences in all physical fitness and body composition *z*-scores across the parent-reported physical activity levels (all *p* < 0.017 and all *p* < 0.001, respectively), as well as, for the fitness index (*p* < 0.001). Our study suggests that in school settings with insufficient resources to objectively assess fitness and body composition, parents-reported physical activity level by means of a single question might provide useful information about these important health markers in preschool children.

## Introduction

Obesity is a worldwide concern that is growing among children and adolescents (Abarca-Gómez et al., 2017), and has even begun to occur in preschool children (Cadenas-Sánchez et al., 2019a). The early onset of obesity seems to be related to the occurrence of associated complications such as metabolic and cardiovascular disorders, and the risks depend partly on the age of onset and on the duration of obesity (Umer et al., 2017; Ho et al., 2019). Particularly worrying is the data coming from the World Health Organization (WHO) whose results demonstrated that in 2014 more than 41 million of children under 5 years old were classified as overweight/obese, and this fact will continue to worsen (World Health Organization, 2016). At European level, a higher prevalence of overweight/obese preschool in the south of Europe was observed, compared to those children from the north (Cadenas-Sanchez et al., 2016b). In addition, in a European cohort of 3120 children aged 2–9 years old (1016 from 2 to 6 years old) objectively measured physical activity was found an important factor to protect against clustering of CVD risk factors in young children (Jiménez-Pavón et al., 2013). At the national level, our group found that 35% of the pre-schoolers examined presented overweight/obesity were already 1.3% morbid obese (Cadenas-Sánchez et al., 2019a). A recent study with a population-based sample of 51,505 children found that the most rapid weight gain occurs between 2 and 6 years of age being those obese at that age also obese adolescents (Geserick et al., 2018). Despite preschool years represents an opportunity for obesity surveillance it is still challenging in the WHO European Region (Jones et al., 2017).

In addition to obesity, a low physical fitness level in childhood/adolescence has been considered a marker of poor health and a risk factor for multiple health outcomes at these ages and later in life (Ortega et al., 2008b; Ruiz et al., 2009). In this context, it has been shown that higher levels of cardiorespiratory fitness during childhood are related to lower risk of becoming overweight/obese across puberty (Ortega et al., 2011). However, such information is not available in preschool children, which could be due, at least partially, to the fact that there was no information about which fitness tests should be used in preschool children. A systematic review searching for any study relating fitness in preschool age with health outcomes, found no studies in this direction (Ortega et al., 2015). Fortunately, over the last years, more evidence has been accumulated on which fitness tests are more feasible, reliable and valid to be used in preschool children, in particular the PREFIT (Assessing FITness in PREschoolers) battery, which will provide new avenues to study the role of fitness in health at these early ages and later in life (Cadenas-Sánchez et al., 2014; Ortega et al., 2015; Sanchez-Delgado et al., 2015; Cadenas-Sanchez et al., 2016a; Mora-Gonzalez et al., 2017). In this context, the PREFIT project group has recently provided for the first time the physical fitness reference standards for preschool children (Cadenas-Sánchez et al., 2019b).

Since obesity and low fitness are important markers of health through the lifespan (Ortega et al., 2018), it is of public health interest to study determinants of these two modifiable risk factors as early as possible, being preschool age a sensitive stage of life. Thus, physical inactivity is a recognized determinant of obesity and low physical fitness, and it would be interesting to explore associations of physical activity with physical fitness and body composition in preschool children. Although objective methods, such as accelerometers, to assess physical activity in young individuals are preferable (Migueles et al., 2017), they have some practical limitations and most importantly they are not feasible under certain circumstances in the school context. Alternatively, parent-reported physical activity levels of their children are an alternative method, and it is of interest to test whether a single question on parents’ rated physical activity level is able for screening a set of important physical fitness and body composition markers in preschool children. If so, this method could be a very useful and economic tool to be used as a predictor of health markers in preschool, similarly to what self-reported PA of older children (10–12 years old) showed in a large epidemiological study (Jiménez-Pavón et al., 2012).

Therefore, the aim of this study is to test whether a single question regarding the physical activity of children, reported by their parents, could be used as a screening tool of objectively measured physical fitness and body composition in preschool children.

## Materials and Methods

### Participants and Study Design

The present study was performed under the framework of the PREFIT project^1^ (Cadenas-Sánchez et al., 2014; Ortega et al., 2015; Sanchez-Delgado et al., 2015; Cadenas-Sanchez et al., 2016a; Mora-Gonzalez et al., 2017; Labayen et al., 2018). Briefly, the aim of the project was to propose a physical fitness test battery of feasible and reliable field-based tests to assess physical fitness in children aged 3–5 years and to report reference values for a better interpretation of physical fitness assessment (Cadenas-Sánchez et al., 2019a,b).

After several methodological studies to define the PREFIT fitness battery (Cadenas-Sánchez et al., 2014; Ortega et al., 2015; Sanchez-Delgado et al., 2015; Cadenas-Sanchez et al., 2016a; Mora-Gonzalez et al., 2017), we set up PREFIT-Spain, aiming to measure physical fitness in a geographically distributed sample of preschool children across Spain. The project involved data collection in 10 cities from the south, center, and north of Spain, gathering a total of 3179 (*M*: 4.60; SD: 0.87) healthy preschool children (52.8% boys and 47.2% girls) aged between 3 and 5 years (Cadenas-Sanchez et al., 2016a). Parents or legal guardians were informed about the purpose of the study and written informed consent was obtained from the parents of all participants. The study protocol was performed in accordance with the ethical standards and was approved by the Review Committee for Research Involving Human Subjects at the University of Granada (n° 845).

### Procedure and Measures

The PREFIT battery comprises the following tests^2^: the PREFIT 20-meters shuttle-run test (PREFIT 20 m SRT) to assess cardiorespiratory fitness, handgrip strength and standing long jump tests to assess muscular strength (upper and lower limbs, respectively), 4 × 10 m SRT to assess speed-agility and one-leg stance test to assess balance (Cadenas-Sanchez et al., 2016a); and weight, height, body mass index (BMI), waist circumference, and waist-to-height ratio to assess body composition indicators. Children were assessed individually in a school-setting by trained evaluators using the following standardized equipment and procedures. Before starting the tests, evaluators performed an example to ensure that the child understood the test correctly.

### Physical Fitness Measures

Cardiorespiratory fitness was assessed using a modified version of the original 20 m shuttle run test (SRT): the PREFIT 20 m SRT (Cadenas-Sánchez et al., 2014; Mora-Gonzalez et al., 2017). Participants had to run back and forth between 2 lines 20 m apart with an audio signal. The test finished when the child failed to reach the end lines concurrent with the audio signal on two consecutive occasions or when the child stops because of exhaustion. The initial stage of the PREFIT 20 m SRT was 6.5 km h^-1^.

The handgrip strength test measures the maximal strength of the upper limb using an analog dynamometer (TKK 5001, Grip-A, Takei, Tokyo, Japan). The test protocol is reported elsewhere (España-Romero et al., 2010). The grip spans were fixed at 4.0 cm (Sanchez-Delgado et al., 2015). The best value of the two attempts for each hand was chosen, and the average of both was registered in kilograms (kg). The standing long jump test assesses the explosive strength of the lower limbs. This test consisted of jumping as far as possible with the feet together and remaining upright. The distance was measured from the take-off line to the point where the back of the heel nearest to the take-off line lands on the ground. The better of two attempts were recorded in centimeters (cm).

The 4 × 10 m SRT was used to assess speed/agility. This test consisted of running and turning as fast as possible between two parallel lines (10 m apart). To make this test easier to understand and perform for preschool children, we did not use sponges to be exchanged when crossing the lines (4 × 10 m), as the original protocol does in older children (Ortega et al., 2008a). Instead, two evaluators were located behind each line and participants had to touch the examiner’s hand and go back at maximum speed. The result was measured with a stopwatch (Tremblay, CHRO 300, Gleizé, France) to the nearest 0.1 s. The best of two attempts was recorded in seconds. A higher score indicates worse performance.

### Body Composition

Weight (kg) was measured without shoes and in light clothing using an electronic scale (SECA Model 869, Hamburg, Germany). Height (cm) was measured in the Frankfort plane without shoes using a stadiometer (SECA Model 213). BMI was calculated based on weight divided by height squared. The cut-off points used to classify weight status categories were those established by World Obesity Federation (WOF)^3^, formerly International Obesity Task Force (IOTF) (Cole and Lobstein, 2012) and by the WHO (de Onis et al., 2007). Waist circumference (cm) was measured at the umbilical location with a non-elastic tape (SECA Model 200) at the end of a normal expiration without the tape compressing the skin. The waist-to-height ratio was calculated as waist circumference divided by height in centimeters. All measures were taken twice and the mean of the two measurements was used for analyses. All measurements were harmonized following a strict protocol to ensure standardization.

### Physical Activity

As an indirect measure of the physical activity level of the children, parents were asked to answer the following question: “Your children’s physical activity level excluding school time is …?” The response options were very low, low, average, high, or very high. The response frequency by categories of parent-reported physical activity levels is shown in Supplementary Figure S1. For analyses, the very low and low categories were merged due to the low response frequency of the very low category.

### Confounders

Paternal and maternal educational was assessed by self-report questionnaire and can be consulted elsewhere (Merino-De Haro et al., 2018). A variable with three categories was calculated for analyses and for each parent: low (no education or primary school education), medium (secondary school education, upper-secondary school education, or technical training), and high (university education). Only, age, gender, and maternal education level were introduced as potential confounders.

### Statistical Analysis

Firstly, we explored which potential confounders were more strongly correlated with the study outcomes. Maternal education was more strongly associated than paternal education with study outcomes and, thus, it was a better predictor. Partial correlation adjusted for potential confounders were used to examine the association of parent-reported physical activity levels with physical fitness and body composition indicators.

To study the association of parent-reported physical activity levels with physical fitness (PREFIT 20 m SRT, handgrip strength test, standing long jump, 4 × 10 m SRT) and body composition outcomes (weight, BMI, waist circumference, waist-to-height ratio), we conducted linear regression analysis inserting the physical fitness and body composition outcomes as dependent variables and parent-reported physical activity levels as independent variable, adjusted for sex, age and maternal education. Additionally, physical fitness and body composition *z*-scores were created and explored using analysis of covariance (adjusted for confounders) with a Bonferroni adjustment according to parent-reported physical activity levels. Also, it was created a fitness index using the average of physical fitness z-scores divided by the number of fitness outcomes (PREFIT 20 m SRT, standing long jump, 4 × 10 m SRT), and explored with an analysis of covariance with a Bonferroni adjustment according to parent-reported physical activity levels. All analyses were performed using the Statistical Package for Social Sciences (IBM SPSS Statistics for Windows, version 21.0, Armonk, NY) and the level of significance was set at *p* < 0.05. Graphics were performed using Sigmaplot version 12.5 for Windows (Systat Software, Inc., San Jose, CA, United States).

## Results

Descriptive characteristics of the physical fitness and body composition indicators of the sample stratified by sex and age groups (3, 4, and 5 years) are presented in Table 1, as means and standard deviation. The response frequency of parent-reported physical activity levels (for the whole sample and by sex) is illustrated in Supplementary Figure S1.

**Table 1:** Descriptive characteristics of the study population (*n* = 3.179).

	All (*n* = 3.179)		Boys (*n* = 1.678)						Girls (*n* = 1.501)					
			3 years		4 years		5 years		3 years		4 years		5 years	
Age (years)	4.60	0.87	3.53	(0.30)	4.50	(0.28)	5.54	(0.34)	3.50	(0.31)	4.53	(0.29)	5.53	(0.32)
Height (cm)	106.93	7.51	99.90	(4.67)	106.91	(4.77)	113.93	(5.49)	98.73	(4.53)	106.01	(4.74)	112.89	(4.85)
**Gender [n (%)]**														
Female	1501	(47.22)							443	(47.48)	509	(47.0)	549	(47.21
Male	1678	(52.83)	490	(52.52)	574	(53.0)	614	(52.79)						
**Maternal education [n (%)]**														
Low	210	(7)	33	(6.98)	27	(5.06)	49	(8.69)	23	(5.45)	33	(6.92)	45	(8.70)
Medium	1382	(46.34)	202	(42.71)	262	(49.06)	272	(48.23)	185	(43.84)	212	(44.44)	249	(48.16)
High	1395	(46.76)	238	(50.32)	245	(45.88)	243	(43.09)	214	(50.71)	232	(48.64)	223	(43.13)
**Physical fitness**														
PREFIT 20 m SRT (laps)	20.02	11.64	12.85	(7.65)	21.19	(9.51)	28.86	(13.05)	10.86	(6.36)	18.56	(8.21)	23.96	(11.45)
Handgrip strength (kg)	7.03	2.48	5.20	(1.69)	7.17	(1.90)	9.27	(2.25)	4.66	(1.58)	6.56	(1.72)	8.37	(1.97)
Standing long jump (cm)	73.63	22.25	57.57	(17.72)	77.93	(16.91)	91.45	(17.36)	50.81	(17.12)	70.60	(16.06)	84.48	(18.10)
4 × 10 m SRT (s)	16.81	2.52	18.82	(2.33)	16.36	(1.76)	14.83	(1.40)	19.77	(2.50)	16.72	(1.59)	15.46	(1.49)
**Fatness**														
Weight (kg)	18.99	3.75	16.55	(2.33)	18.78	(2.95)	21.62	(4.04)	16.12	(2.16)	18.52	(2.93)	21.16	(3.59)
BMI (kg/m^2^)	16.48	1.76	16.53	(1.50)	16.36	(1.63)	16.55	(2.03)	16.48	(1.33)	16.41	(1.80)	16.54	(2.01)
Waist circumference (cm)	53.16	5.02	50.88	(3.80)	52.59	(4.24)	54.95	(5.57)	51.24	(3.86)	53.29	(4.88)	55.19	(5.47)
Waist-to-height ratio	0.50	0.04	0.51	(0.03)	1.49	(0.03)	0.48	(0.04)	0.52	(0.03)	0.50	(0.04)	0.49	(0.04)

*SD, standard deviation; SRT, shuttle run test; BMI, body mass index. Values are presented as mean and standard deviation.*

The results show the association of parent-reported physical activity levels between physical fitness and body composition measures after adjusting for sex, age and maternal education in preschool children. Children whose parents reported to have higher physical activity levels had a higher level of physical fitness in all indicators (β_range_ = -0.094 to 0.113, and *p*-value from 0.020 to <0.001) (Table 2). Otherwise, it was observed a negative association between parent-reported physical activity levels and all body composition indicators after adjusting for sex, age and maternal education (β_range_ = -0.058 to -0.113, all *p* < 0.001). The results showed similar trends for physical fitness and body composition of children with different age and sex (see Supplementary Tables S1 and S2, respectively).

**Table 2:** Associations of parents-reported physical activity levels with objectively measured physical fitness and body composition in preschool children (*n* = 3.179).

	β	*B*	CI (95%)	*p*
PREFIT 20 m SRT (laps)	0.113	1.714	(1.252, 2.176)	<0.001
Handgrip strength (kg)	0.031	0.100	(0.016, 0.185)	0.020
Standing long jump (cm)	0.102	2.971	(2.174, 3.768)	<0.001
4 × 10 m SRT (s)^∗^	-0.094	-0.309	(-0.394, -0.224)	<0.001
Weight (kg)	-0.058	-0.284	(-0.429, -0.139)	<0.001
BMI (kg/m^2^)	-0.097	-0.223	(-0.307, -0.139)	<0.001
Waist circumference (cm)	-0.100	-0.658	(-0.883, -0.433)	<0.001
Waist-to-height ratio	-0.113	-0.006	(-0.008, -0.004)	<0.001

*SRT, shuttle run test; BMI, body mass index; β, beta standardized coefficients; B, beta unstandardized coefficients; CI, confidence interval (upper, lower). Parent-reported physical activity levels were considered as a predictive variable and physical fitness outcomes (PREFIT 20 m SRT, handgrip strength test, standing long jump and 4 × 10 m SRT) and body composition outcomes (weight, BMI, waist circumference and waist-to-height ratio) were considered as dependent variables. The analyses were adjusted by age, sex, and maternal education. ^∗^Lower values indicate better performance.*

**FIGURE 1:**
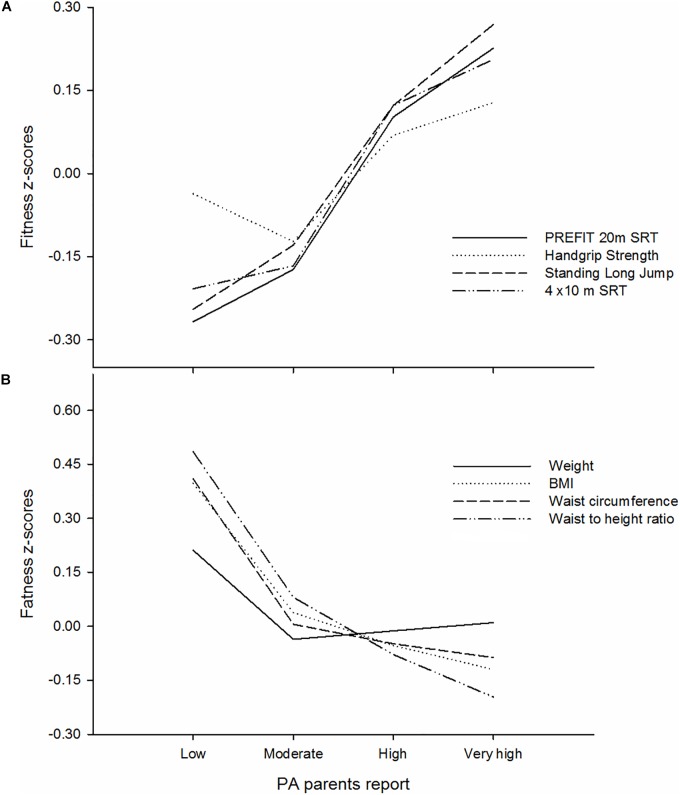
Differences of objectively measured physical fitness *z*-scores **(A)** and body composition *z*-scores **(B)** across parents-reported physical activity levels in preschool children (*n* = 3.179). SRT, shuttle run test; BMI, body mass index; PA, physical activity. The analyses were adjusted by age, sex, and maternal education.

The results show the differences in objectively measured physical fitness *z*-scores (panel A) and body composition *z*-scores (panel B) across parent-reported physical activity levels. The results showed significant differences in all physical fitness and body composition *z*-scores across the parent-reported physical activity levels (all *p* < 0.017 and all *p* < 0.001, respectively) (Figure 1). There were pairwise significant differences in PREFIT 20 m SRT, standing long jump and the 4 × 10 m SRT between each category of the parent-reported physical activity levels after a Bonferroni adjustment (all *p* < 0.038). There were pairwise significant differences in each category of the parent-reported physical activity levels after a Bonferroni adjustment and all body composition *z*-scores between (all *p* < 0.036). However, these pairwise significant differences were not seen between the high and very high categories in neither physical fitness *z*-scores nor body composition *z*-scores (*p*_range_ = 0.059 to 0.934 and *p*_range_ = 0.128 to.355, respectively).

**FIGURE 2:**
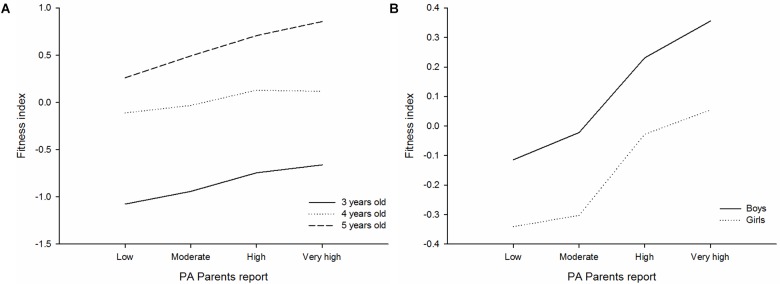
Differences of physical fitness index across parent-reported physical activity levels, by age **(A)** and gender **(B)** in preschool children (*n* = 3.179). SRT, shuttle run test; PA, physical activity. The analyses were adjusted by age, sex, and maternal education.

The results show the differences in physical fitness index across parent-reported physical activity levels, by age (Figure 2A) and gender (Figure 2B). The results showed significant differences in the fitness index across the parent-reported physical activity levels (*p* < 0.001). There were pairwise significant differences in the fitness index between each category of parent-reported physical activity levels after a Bonferroni adjustment (all *p* < 0.001). However, these pairwise significant differences were not seen between the high and very high categories (*p* = 0.102). No interaction effect was seen for age groups (*p* = 0.233) nor gender (*p* = 0.722).

## Discussion

This study provides evidence supporting that a single and very simple question of physical activity reported by parents can significantly estimate the physical fitness and body composition of preschool children (aged 3–5 years). More specifically, our findings support that: (1) parent-reported physical activity levels are positively associated with objectively measured physical fitness, as measured by means of the PREFIT 20 m SRT, handgrip strength, standing long jump and the 4 × 10 m SRT in children aged 3–5 years; (2) parent-reported physical activity levels are negatively related to body composition indicators, as measured by weight, BMI, waist circumference and waist-to-height ratio in children aged 3–5 years; and (3) there are pronounced differences in all physical fitness and body composition *z*-scores, as well as, in the fitness index across each category of the parent-reported physical activity levels in children aged 3–5 years.

This study shows a positive association between parent-reported physical activity levels and the PREFIT 20 m SRT. These results are in agreement with previous findings in the adult population (Emaus et al., 2010; Minder et al., 2014). For instance, Murphy et al. (1988) who found that a reported physical activity single question was associated with maximal oxygen consumption, measured by a more valid and reliable technique. Likewise, Aadahl et al. (2007) showed that reported physical activity was related to objectively measured cardiorespiratory fitness. Nevertheless, in our study inconsistent associations were seen between parent-reported physical activity levels and handgrip strength (see Supplementary Table S1). In fact, when analyses were undertaken by age group only was seen as an association in those older preschool children. This result does not concur with those found in other population of adults where self-reported measures of physical activity were correlated with handgrip strength (Leblanc et al., 2015). In fact, a recent systematic review has found inconsistent associations between muscular fitness and self-reported physical activity in children and adolescents (Smith et al., 2019). Otherwise, our data showed that parent-reported physical activity levels correlate strongly with lower body strength as measured by means of standing long jump. In this sense, it can be hypothesized that uncontrolled variables (fat-free mass, fat mass) in our sample might explain the discrepancies between the associations of parent-reported physical activity levels and the distinct components of the muscular strength (Artero et al., 2010).

A negative association between parent-reported physical activity levels and body composition indicators was found in this investigation. To our knowledge, there are no studies that analyze this association in preschool children, and that’s why this study opens a new window in order to estimate childhood obesity based on physical activity information reported by parents. Recent studies have found that higher moderate-to-vigorous physical activity (MVPA) and/or vigorous physical activity of preschool children are associated with better body composition (Leppänen et al., 2016, 2017; Fang et al., 2017). In this context, a self-reported methodology to assess physical activity levels of preschool children has not been recommended (Oliver et al., 2007), however, parents might do with somewhat validity (Pate et al., 2010). Taking this evidence together, it can be suggested that parent-reported physical activity levels of preschool children might be an alternative to estimate objectively measured body composition indicators since physical activity is associated with body composition at an early age.

Our results also showed that there was a pronounced difference between children with very low/low physically active (who had worse physical fitness and body composition) and the rest of the categories with higher reported physical activity; however, the question did not discriminate so accurately between those preschool children reported by their parents as being moderate, high and very high active. This finding suggests that our question might be more useful in more extreme cases (between very low/low and high/very high active). In this context, the question proposed in this investigation could be used to perform the first screening with large samples of preschool in order to detect the most extreme cases with a marked risk of worse physical fitness and body composition. Thus, objective and direct measures could be administered only in those participants who have already been detected at higher risk. Therefore, this single question about PA levels would serve to identify children at a higher risk of low fitness and worse body composition avoiding the complications associated with direct and objectively measurement.

### Limitations and Strengths

This research has several limitations. The cross-sectional nature of the study and the use of an indirect measure of physical activity levels should be considered with caution. In addition, despite all associations of parent-reported physical activity levels between physical fitness and body composition were statistically significant the standardized regression coefficients were small. Likewise, the reliability of the single question has not been yet proved, which could be another drawback. To our knowledge, this large, population-based study on preschool children is the first that covers geographically most of the regions of Spain. Also, the use of a quick and single question able to identify vital markers of physical health at such early ages should be considered as something powerful.

## Conclusion

Our study suggests that a single question of parent-reported physical activity levels might provide meaningful information to identify children at a higher risk of important physical health markers such as physical fitness and body composition in preschool children. Therefore, a single question could be an alternative for a physical health screening at early ages, especially for epidemiological and intervention studies with insufficient resources or even in school settings where objective methods cannot be applied.

## Data Availability

The datasets generated for this study are available on request to the corresponding author.

## Ethics Statement

The study protocol was performed in accordance with the ethical standards and was approved by the Review Committee for Research Involving Human Subjects at the University of Granada (number 845).

## Author Contributions

CC-S and FBO conceptualized and designed the study. CC-S organized the database. AM-M, CC-S, FBO, and JV-C carried out the statistical analysis. PP, AM-M, JC, PB, FBO, and JV-C wrote the first draft of the manuscript. PP, AM-M, JC, PB, FBO, and JV-C wrote the sections of the manuscript. All authors contributed to the manuscript revision, read, and approved the final version of the manuscript.

## Conflict of Interest Statement

The authors declare that the research was conducted in the absence of any commercial or financial relationships that could be construed as a potential conflict of interest. The handling Editor is currently editing co-organizing a Research Topic with two of the authors, AM-M and PP, and confirms the absence of any other collaboration.

## Footnotes

1 http://profith.ugr.es/prefit

2 http://profith.ugr.es/recursos-prefit?lang=en

3 http://www.worldobesity.org/
