# Supplementary material for: A Single Question of Parent-Reported Physical Activity Levels Estimates Objectively Measured Physical Fitness and Body Composition in Preschool Children: The PREFIT Project
Source: Front Psychol. 2019 Jul 10;10:1585. doi: 10.3389/fpsyg.2019.01585 (PMC6635596; doi:10.3389/fpsyg.2019.01585)
Supplement: Supplementary file 1 [file Table_1.DOCX]

Supplementary Material

**Table S1.** Associations of parent-reported physical activity levels with objectively measured physical fitness in preschool children (n= 3.179).

|  | 3 years  (n=933) | | | |  | 4 years  (n=1.083) | | | |  | | 5 years  (n=1.163) | | | | |
| --- | --- | --- | --- | --- | --- | --- | --- | --- | --- | --- | --- | --- | --- | --- | --- | --- |
|  | β | B | CI (95%) | p |  | β | B | CI (95%) | p |  | β | | B | CI (95%) | p |  |
| PREFIT 20m SRT (laps) | .124 | 1.239 | (.602, 1.877) | <.001 |  | .084 | .990 | (.263, 1.717) | .008 |  | .170 | | 2.675 | (1.765, 3.584) | <.001 |  |
| Handgrip strength (kg) | .052 | .118 | (-.026, .262) | .107 |  | .003 | .006 | (-.134, .147) | .929 |  | .056 | | .154 | (.005, .304) | .043 |  |
| Standing long jump (cm) | .136 | 3.340 | (1.829, 4.852) | <.001 |  | .085 | 1.879 | (.573, 3.185) | .005 |  | .160 | | 3.641 | (2.326, 4.956) | <.001 |  |
| 4×10m SRT (s) | -.125 | -.418 | (-.624, -.211) | <.001 |  | -.125 | -.265 | (-.392, -.137) | <.001 |  | -.143 | | -.265 | (-.370, -.160) | <.001 |  |

SRT: shuttle run test. β: Beta standardized coefficients. B: Beta unstandardized coefficients. CI=confidence interval (upper, lower). Physical fitness outcomes (PREFIT 20m SRT, handgrip strength test, standing long jump and 4x10m SRT) were considered as dependent variables and parents-reported physical activity levels was considered as a predictive variable. The analyses were adjusted by maternal education.

**Table S2.** Associations of parent-reported physical activity levels with objectively measured body composition in preschool children (n= 3.179).

|  | 3 years  (n=933) | | | |  | 4 years  (n=1.083) | | |  |  | 5 years  (n=1.163) | | | |
| --- | --- | --- | --- | --- | --- | --- | --- | --- | --- | --- | --- | --- | --- | --- |
|  | β | B | CI (95%) | p |  | β | B | CI (95%) | p |  | β | B | CI (95%) | p |
| Weight (kg) | .014 | .044 | (-.161, .248) | .676 |  | -.102 | -.388 | (-.620, -.156) | .001 |  | -.087 | -.425 | (-.708, -.142) | .003 |
| BMI (kg/m^2^) | -.041 | -.080 | (-.213, .053) | .236 |  | -.119 | -.268 | (-.409, -.126) | <.001 |  | -.111 | -.286 | (-.440, -.133) | <.001 |
| Waist circumference (cm) | -.030 | -.161 | (-.519, .197) | .378 |  | -.105 | -.632 | (-.006, -.258) | .001 |  | -.145 | -1.019 | (-1.432, -.606) | <.001 |
| Waist-to-height ratio | -.075 | -.003 | (-.006, .000) | .024 |  | -.095 | -.005 | (-.008, -.002) | .003 |  | -.162 | -.009 | (-.012, -.005) | <.001 |

BMI: body mass index. β: Beta standardized coefficients. B: Beta unstandardized coefficients. CI=confidence interval (upper, lower). Body composition outcomes (weight, BMI, waist circumference and waist-to-height ratio) were considered as dependent variables and parent-reported physical activity levels was considered as a predictive variable. The analyses were adjusted by maternal education.

**Figure S1.** Response frequency by categories of parent-reported physical activity levels in preschool children (n= 3.179).
